# Comparison of Geographical Traceability of Wild and Cultivated *Macrohyporia cocos* with Different Data Fusion Approaches

**DOI:** 10.1155/2021/5818999

**Published:** 2021-07-21

**Authors:** Li Wang, Qinqin Wang, Yuanzhong Wang, Yunmei Wang

**Affiliations:** ^1^Quality Standards and Testing Technology Research Institute, Yunnan Academy of Agricultural Sciences, Kunming 650205, China; ^2^College of Agronomy and Biotechnology, Yunnan Agricultural University, Kunming 650201, China; ^3^The First Affiliated Hospital of Yunnan University of Traditional Chinese Medicine, Kunming 650021, China; ^4^Medicinal Plants Research Institute, Yunnan Academy of Agricultural Sciences, Kunming 650200, China

## Abstract

Poria originated from the dried sclerotium of *Macrohyporia cocos* is an edible traditional Chinese medicine with high economic value. Due to the significant difference in quality between wild and cultivated *M. cocos*, this study aimed to trace the origin of the fungus from the perspectives of wild and cultivation. In addition, there were quite limited studies about data fusion, a potential strategy, employed and discussed in the geographical traceability of *M. cocos*. Therefore, we traced the origin of *M. cocos* from the perspectives of wild and cultivation using multiple data fusion approaches. Supervised pattern recognition techniques, like partial least squares discriminant analysis (PLS-DA) and random forest, were employed in this study using. Five types of data fusion involving low-, mid-, and high-level data fusion strategies were performed. Two feature extraction approaches including the selecting variables by a random forest-based method—Boruta algorithm and producing principal components by the dimension reduction technique of principal component analysis—were considered in data fusion. The results indicate the following: (1) The difference between wild and cultivated samples did exist in terms of the content analysis of vital chemical components and fingerprint analysis. (2) Wild samples need data fusion to realize the origin traceability, and the accuracy of the validation set was 95.24%. (3) Boruta outperformed principal component analysis (PCA) in feature extraction. (4) The mid-level Boruta PLS-DA model took full advantage of information synergy and showed the best performance. This study proved that both geographical traceability and optimal identification methods of cultivated and wild samples were different, and data fusion was a potential technique in the geographical identification.

## 1. Introduction

*Macrohyporia cocos* is a wood-decay fungus in the Polyporaceae family. It transforms the wood of pine trees into medicinal products, which could treat various edemas, invigorate the spleen function, and calm the mind. The sclerotium of *M. cocos*, called Poria, is one of the most widely used raw materials of Chinese herbal compound preparations. The Chinese Pharmacopoeia (version 2015) records over one hundred types of prescriptions including Poria. Moreover, the National Health Commission of the People's Republic of China has approved that this fungus could be used for food. Plenty of Poria-based skin cosmetics like facial masks have been produced and used. Present investigation shows that this fungus displays not only anticancer [1, 2] but anti-inflammatory [3, 4], antihyperlipidemic [5], renoprotective [6], and hepatoprotective [7] properties thanks to both its polysaccharides profile and the presence of terpenes. In other words, Poria has shown high economic value and medicinal value.

Geographical traceability has always been a significant issue for quality assurance, not only for natural medicine but also for food. Many laws have been legislated for protecting part of geographical origins, such as China Protected Geographical Indication Products and European Protected Designation of Origin [8]. It is therefore of great importance to find a suitable analytical method to identify the geographical origin for guaranteeing the quality as well as the reasonable utilization. Nowadays, various analytical methods, such as liquid chromatography (LC) and its hyphenated techniques (HPLC-PAD and UHPLC-QTOF-MS/MS) [9, 10], mid- and near-infrared spectroscopy [11, 12], and ultraviolet spectroscopy [13] individually and jointly [14, 15], have been used for the geographical traceability of the species, wherein data fusion strategies show great potentiality in this aspect.

By means of combining the outputs of multiple complementary information regarding objects to exploit the synergies of information, data fusion strategies have more opportunities to achieve an accurate characterization than single pieces of data [16], which have been applied in the fields of food, beverage, and medicine [17–20]. Wang et al. [14] carried out the geographical authentication of cultivated *M. cocos* by liquid chromatography and infrared spectroscopy combined with data fusion, and the results witnessed that the performance of low-level data fusion strategy preceded that of the single techniques. Li and Wang [15] performed the comparison of *M. cocos* raw materials using ultraviolet (UV) and Fourier transform infrared (FTIR) spectroscopy data fusion, and it was found that the differences in growth patterns were larger than those in collection regions, whereas only a few types of data fusion methods were studied and discussed. Additionally, growth patterns have influences on the chemical composition of both inner part and epidermis of the species [21], and traditionally wild species were more popular than cultivated ones; however, they fail to discuss and distinguish the geographical origins in terms of wild and cultivated separately. Accordingly, the purpose of the study was to trace the origin of *M. cocos* from the perspectives of wild and cultivation using multiple data fusion methods.

Five types of data fusion methods involving low-, mid-, and high-level data fusion strategies were studied: one low-level, two mid-level, and two high-level. Each level fusion had its advantages. Low-level fusion was characterized by its easy implementation. Mid-level data fusion could save the computation time because its step of feature extraction significantly reduced the data dimensionality [22]. The merit of high-level one was that when there was a new block of data which revealed new features that were useful for deciphering the objects, it could be added to the classification decision instantly to increase the versatility of decision process [16]. Feature extraction could be also used in high-level fusion.

According to our previous experiences regarding origin identification of cultivated Poria, the inner part with larger output was more efficient than the epidermis, and liquid chromatograms at 242 nm and FTIR spectra outperformed other techniques [14]. Therefore, the fingerprints of FTIR spectroscopy and liquid chromatography under the wavelength of 242 nm were chosen to characterize the inner part of wild and cultivated samples.

In this study, five data fusion methods combined with two multivariate classification approaches of partial least squares discriminant analysis (PLS-DA) and random forest (RF) have been applied to take advantage of the synergistic effect of the information obtained from FTIR and LC. In particular, two feature extraction approaches including the selecting variables by a RF-based method—Boruta algorithm and producing principal components (PCs) by the dimension reduction technique of principal component analysis (PCA)—were considered in data fusion. The quality, geographical traceability, and optimal identification methods of cultivated and wild samples were compared. The results of this study may improve the current knowledge and pave the way for further development and utilization of this fungus.

## 2. Materials and Methods

### 2.1. Chemicals and Sample Preparation

HPLC-grade acetonitrile was purchased from Thermo Fisher Scientific (Fair Lawn, NJ, USA). Formic acid was purchased from Dikma Technologies (Lake Forest, CA, USA). Purified water was purchased from Guangzhou Watsons Food & Beverage Co., Ltd. (Guangzhou, China). Other chemicals and reagents were of analytical grade. The standard compounds (pachymic acid, dehydropachymic acid, poricoic acid A, dehydrotrametenolic acid, and 3-epidehydrotumulosic acid) (purity ≥ 98%) were supplied by Beijing Keliang Technology Co., Ltd. (Beijing, China). Dehydrotumulosic acid (purity ≥ 96%) was purchased from ANPEL Laboratory Technologies Inc. (Shanghai, China). The concentration ranges of standard solutions prepared for each analyte were the following (mg·L^−1^): dehydrotumulosic acid: 5.00–999; poricoic acid A: 0.22–6730; 3-epidehydrotumulosic acid: 1–100; dehydropachymic acid: 2.4–480; pachymic acid: 10.3–1240; and dehydrotrametenolic acid: 0.49–2450.

Both wild and cultivated samples (123) were collected from Yuxi, Pu'er, Dali, Chuxiong, and Baoshan of Yunnan Province, China. The detailed information was showed in Table S1. All mature samples were collected from July to September. All of the samples were identified as *Macrohyporia cocos* (Schwein.) I. Johans. & Ryvarden by Professor Yuanzhong Wang (Institute of Medicinal Plant, Yunnan Academy of Agricultural Sciences, Kunming, China). For fresh sclerotium, the attached soil was brushed away and washed by tap water. Then, the samples were air-dried in the shade with good ventilation. The dark epidermis was removed, and white inner part was powdered afterwards for analysis. The powder was screened with 60-mesh sieve. All samples were preserved in polyethylene resealable bags for further analysis.

Then, on the one hand, accurately weighed powder (0.5000 ± 0.0001 g) was ultrasonically dissolved in 2.0 mL methanol for 40 min. The extract was filtered through a 0.22 *μ*m membrane filter. The filtrates collected in auto sampler were injected into the LC system for analysis. On the other hand, sample powder was used directly for attenuated total reflectance FTIR spectra acquisition.

### 2.2. Chromatographic Analysis

LC analyses were performed with an ultra-fast liquid chromatography system (Shimadzu, Japan) equipped with a UV detector, a thermostatic column compartment, an autosampler, a degasser, and binary gradient pumps. The separation was carried out on an Inertsil ODS-HL HP column (3.0 × 150 mm, 3 *μ*m) operated at 40°C. The mobile phase consisted of acetonitrile (A) and 0.05% formic acid (B). The flow rate was kept at 0.4 mL·min^−1^, and the injection volume was set at 7 *μ*L. The signals were acquired at 242 and 210 nm. Before use, the mobile phase constituents were degassed by ultrasonication and filtered through a 0.2 *μ*m filter. The samples were eluted with the following gradient: 40% A (0.00 min⟶25.00 min), 40%⟶69% A (25.00 min⟶52.00 min), 69%⟶72% A (52.00 min⟶56.00 min), 72%⟶78% A (56.00 min⟶58.00 min), 78%⟶90% A (58.00 min⟶58.01 min), and 90% A (58.01 min⟶60 min). Each run was followed by an equilibration period of three minutes with initial conditions (40% A and 60% B).

### 2.3. Spectral Acquisition

A FTIR spectrometer (Perkin Elmer, USA) equipped with deuterated triglycine sulfate (DTGS) detector and attenuated total reflectance (ATR) sampling accessory was used to record FTIR spectra. The resolution and scan range were set as 4 cm^−1^ and 4000–650 cm^−1^. Each sample was scanned sixteen times successively. The air spectrum was recorded for background correction. This experiment was implemented under a constant temperature (25°C) and humidity (30%) condition.

### 2.4. Data Processing and Analysis

#### 2.4.1. Pretreatment of Chromatograms and Spectra

The retention time of chromatograms would be affected by time and other factors. For this reason, the correlation-optimized warping algorithm [23] was used for correcting the retention time shifts among samples. In order to save computation time, the corrected chromatographic data was reduced by taking one in every three points without affecting the chromatographic features. Besides, all the original FTIR spectra were subjected to advanced ATR correction using OMNIC 9.7.7 (Thermo Fisher Scientific, USA). The spectral bands at 4000–3700 cm^−1^ and 2670–1750 cm^−1^ had noise; therefore, the variables in both ranges were abandoned. Because chromatograms and spectra contained overlapped peaks and baseline shifts, Savitzky–Golay (SG) polynomial second-derivative filter (second-order polynomial and 15-point window) was carried out to highlight slight differences and eliminate the interference of baseline drift. Particularly, the deletion of spectral variable was performed after SG polynomial second-derivative preprocessing.

The size of data matrix (*m* × *n*) was built to describe the change in variable numbers, where *m* represented the number of samples and *n* represented the retention time of chromatogram or the wavenumber of spectrum. Take wild samples for example; the initial chromatographic matrix (61 × 7201) was transformed as (61 × 2387) after pretreatment, and the raw spectral matrix (61 × 1737) was changed as (61 × 1097). The processed data matrixes were then used for PLS-DA, random forest or data fusion.

#### 2.4.2. Data Fusion

**Figure 1:**
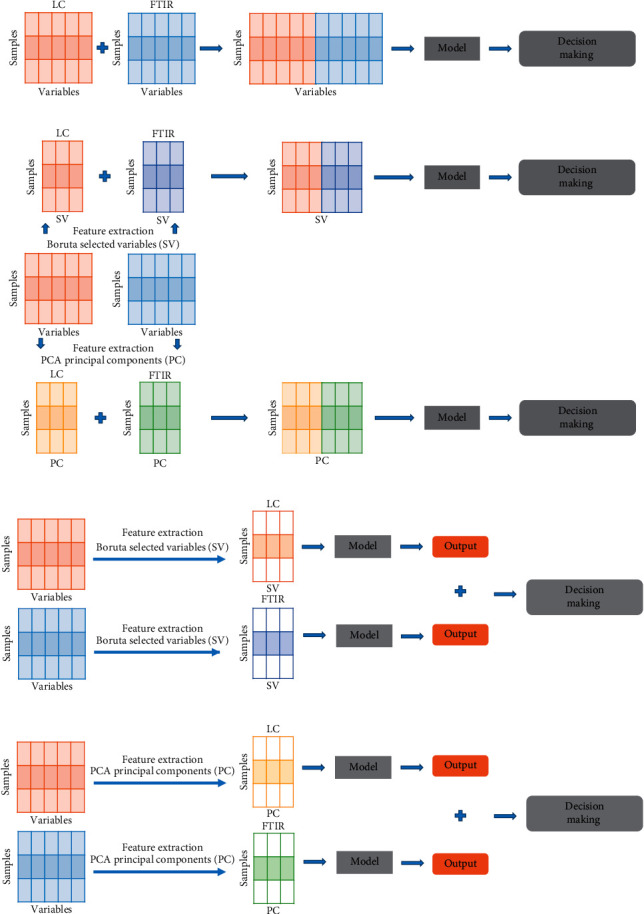
The schemes of data fusion strategies. (a) Low-level data fusion. (b) Mid-level data fusion. (c) High-level data fusion.

Data fusion strategies, which integrated the outputs of multiple complementary information, were expected to obtain more accurate characterization than single information. In the process of data fusion, it was the LC and FTIR data of the same sample that were combined. Three levels of data fusion were studied: low-, mid-, and high-level. Low-level fusion was conceptual simplicity and easy implementation. Several preprocessed datasets were straightforward concatenated into a matrix, whose variables number was equal to the sum of the variables number from each dataset. The important step of mid-level fusion was to extract relevant features from each dataset independently, then concatenating them into a new matrix employed for multivariate analysis. In high-level, each dataset was calculated by a model, and the outputs of each individual model were integrated to obtain final judgement using the fuzzy set theory [24]. In brief, the final decision depended on the result of a majority vote of four fuzzy aggregation connective operators (maximum, minimum, average, and product). The specific schemes of the data fusion process in this study are represented in Figure 1.

Feature extraction could save the computation time and improve the accuracy in practical model building [25], and two extracting features methods were used: (1) PCs extraction employing the dimension reduction technique of principal component analysis. As new variables with a small number, PCs almost described a large proportion of the original information [26]. The number of PCs was determined by 7-fold cross-validation procedure of SIMCA software. (2) Variable selection applying the Boruta algorithm. Boruta was an RF-based feature extraction method, which unbiasedly and stably selected important and nonimportant variables from an information system [25]. The variables marked with the decision of tentative and confirmed were regarded as important features and extracted.

#### 2.4.3. Chemometrics

Chemometrics approaches were playing an essential role in the fields of food and pharmaceutical sciences. Supervised pattern recognition techniques, like PLS-DA and RF, were employed in this study. Once a classification model was built, the membership of a sample of unknown class to predefined classes could be recognized. Partial least squares discriminant analysis (PLS-DA) was a wildly used linear classification method combining the properties of partial least squares regression with classification technique [27, 28]. As the primary parameter, the number of latent variables was carried out based on 7-fold cross-validation procedure. The important variables to recognize categories correctly could be identified by the variable importance for the projection (VIP) [29].

Random forest (RF) was a method of ensemble learning based on decision of classification or regression trees [30, 31]. When building each individual tree, approximately two-thirds of samples in the calibration set generated a training set, and other one-third of samples were used to obtain an unbiased estimate of the classification error internally. The one-third of samples were also called out-of-bag (OOB) samples. As two crucial tuning parameters in the establishment of random forest model, the number of trees (n_tree_) and *mtry* were chosen depending on OOB classification error. The operational steps were roughly divided into the following four steps. Firstly, a dataset processed by Kennard–Stone algorithm [32] was imported. Secondly, we selected the optimal n_tree_ depending on the lower OOB classification error values of total classes considering each class at the same time, and the initial value of n_tree_ was tested with 2000. Thirdly, the optimal *mtry* was searched in the range of default value of *mtry* (square root of the number of variables) plus minus 10 with step by step [33]. If there were several *mtry*'s with lowest OOB classification error, the one that was closer to the default value came first. Finally, the RF model was built by using the selected n_tree_ and *mtry*.

#### 2.4.4. Evaluation of Model Performance

For assessing the performance of model, the calibration and validation sets were divided at the ratio of 2 : 1 employing Kennard–Stone algorithm. The calibration set was applied to build a model and the validation set was employed to obtain an estimate of the model practicability from an external perspective. In general, if the performance of calibration set is far higher than that of validation set, it shows the possibility of overfitting, that is, diminishing generalization ability of model, which should be avoided.

Additionally, the efficiency and total accuracy rate were as synthetic parameters to evaluate the classification performance. The higher were the values of these parameters, the better was the model performance. The equation of efficiency was shown as follows [34], where TP (true positive) was the number of correctly identified samples in target positive class and TN (true negative) was the number of correctly identified samples in target negative class. By analogy, FP (false positive) and FN (false negative) represented the number of incorrectly identified samples in positive and negative classes, respectively. Total accuracy rate was the percentage of correctly identified samples in the samples from all the classes.

(1) $$\begin{array}{c} \text{Efficiency}=\sqrt{\frac{\text{TP}\times \text{TN}}{\left(\text{TP}+\text{FN}\right)\times \left(\text{TN}+\text{FP}\right)}}. \end{array}$$

#### 2.4.5. Software

SIMACA-P^+^ (version 13.0, Umetrics, Sweden) was used for PCA, PLS-DA, and SG polynomial second-derivative preprocessing. The random forest and Boruta were unfolded using *R* package (version 3.4.3). The correlation optimized warping and Kennard–Stone algorithms were performed by MATLAB software (version R2017a, MathWorks, USA). Contents of five target compounds were statistically analyzed by one-way analysis of variance at *P* < 0.05 using SPSS software (version 21.0, IBM Corporation, USA).

## 3. Results

### 3.1. Pretreatment of Chromatograms and Spectra

**Figure 2:**
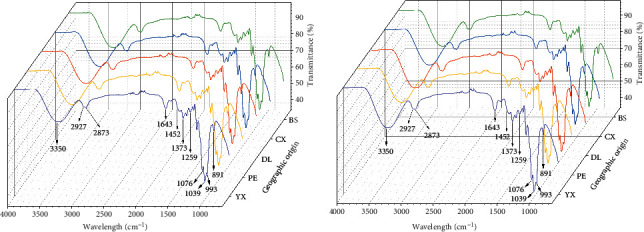
The original FTIR spectra of wild (a) and cultivated (b) *M. cocos* samples from five geographical origins.

**Figure 3:**
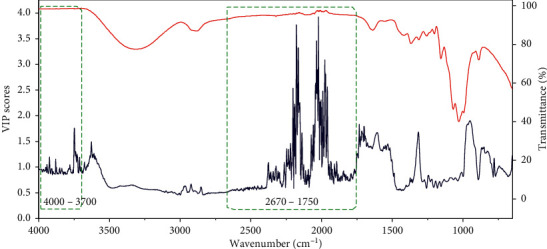
The VIP scores (dark line) extracted from the PLS-DA model of FTIR data of wild samples, appended with the instrumental signal recorded on a sample (red line). the variables in green rectangle areas are chosen to delete.

FTIR spectra of *M. cocos* (Figure 2) presented the structural information of mixture, including the bands of C=O, C=CH_2_, C-O, C-OH, O-H, C-C, and C-H. The variables in the bands of 2670–1750 cm^−1^ and 4000–3700 cm^−1^ were excluded after spectral pretreatment. The specific reasons were as follows: firstly, there was no absorption in these regions. Secondly, according to the usage of VIP, if the VIP score of one wave number was more than one, it was customarily considered helpful to recognize each class correctly [29]. As shown in Figure 3 that the VIP plot of PLS-DA of FTIR data regarding wild samples, the VIP values in the regions of 2670–1750 and 4000–3700 cm^−1^ (rectangle areas in Figure 3) were irregular and almost more than one, which accounted for the presence of chemical interference. Horn et al. [35] reported that the signal of 2670–1750 cm^−1^ was caused by crystal material of ATR accessory.

**Figure 4:**
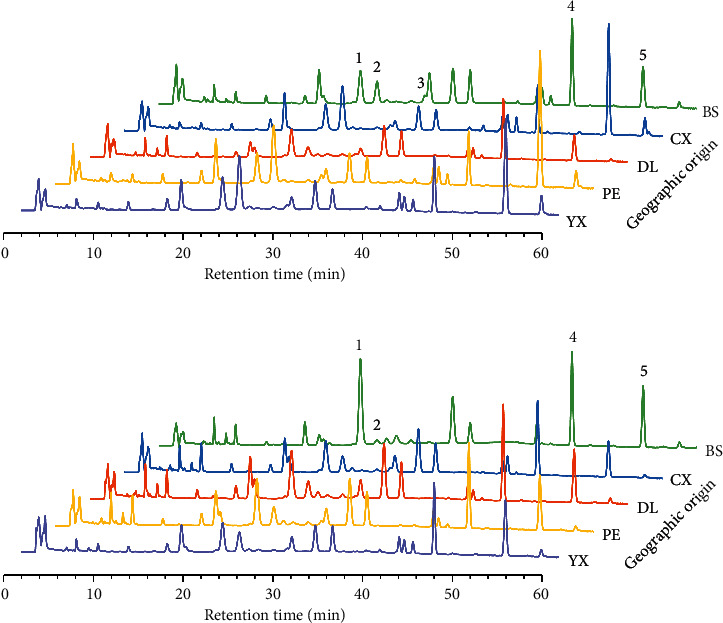
The chromatograms of wild (a) and cultivated (b) *M. cocos* samples from five geographical origins. Peaks 1–5 are dehydrotumulosic acid, poricoic acid A, 3-epidehydrotumulosic acid, dehydropachymic acid, and dehydrotrametenolic acid.

By the way of comparing with the retention time of reference substances, the retention order of the *M. cocos* constituents was found to be dehydrotumulosic acid, poricoic acid A, 3-epidehydrotumulosic acid, dehydropachymic acid, pachymic acid, and finally dehydrotrametenolic acid. Pachymic acid showed patently in the chromatogram of 210 nm, and others existed in that of 242 nm (Figure S1). Based on the previous work, the precision, stability, repeatability, and recovery of chromatographic method were evaluated [14] using dehydrotumulosic acid, poricoic acid A, dehydropachymic acid, pachymic acid, and dehydrotrametenolic acid, which owned good degree of separation. The results showed that all of the relative standard deviation values were lower than 5.95% and recovery rates were from 96.32% ranging to 106.4%, indicating the method was reliable. The correlation coefficients were higher than 0.99 for the calibration curves of the five reference compounds; therefore, the method could be deemed accurate. The limit of quantification (LOQ) and limit of detection (LOD) (determined by diluting continuously standard solution until the signal-to-noise ratios reached 10 and 3, resp.), regression equations, correlation coefficients, and linear ranges of five reference compounds were shown in Table S2. The fingerprints of 242 nm (Figure 4), which presented relatively smooth baseline, would be chosen for further analysis.

Both FTIR and LC were pretreated by SG polynomial second-derivative method to highlight fingerprint differences and eliminate the interference of baseline drift. Compared to raw data, the PLS-DA models processed by SG polynomial second-derivative presented higher accuracy and efficiency values (Table S3), which indicated this method worked.

### 3.2. Comparison of Cultivated and Wild Samples

**Figure 5:**
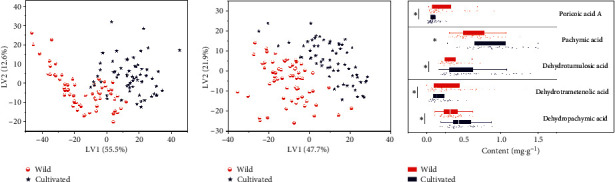
The PLS-DA scores scatter plot based on LC (a) and FTIR (b) data and the box-plot (c) of five triterpene acids contents (mg·g^−1^) regarding wild and cultivated samples. ^*∗*^*P* < 0.05.

The PLS-DA was performed using wild and cultivated samples as class ID. From the scores scatter plots of two dimensions regarding all of samples (Figures 5(a) and 5(b)), it could be easily found that the wild samples were located in the bottom left, and the cultivated ones were located in the top right corner, indicating the large difference among them. Moreover, wild samples were significantly different from cultivated ones in the contents of five vital chemical components (Figure 5(c)) (*P* < 0.05). Accordingly, the cultivated and the wild samples should be performed for origin identification separately.

In addition, the content of terpenoids in the wild and cultivated *M. cocos* differs significantly in the same area. The content of dehydrotumulosic acid, poricoic acid A, and dehydropachymic acid of cultivated *M. cocos* was higher than that of wild samples, and the content of pachymic acid and dehydrotrametenolic acid of wild *M. cocos* was higher than that of cultivated samples in Pu'er area. In general, the quality of cultivated *M. cocos* samples from Chuxiong, Dali, and Baoshan is slightly higher than that of wild samples. The quality of cultivated *M. cocos* in Baoshan is the best, which is suitable for large-scale planting. Yuxi may be the geographic source for screening excellent wild *M. cocos* germplasm resources.

### 3.3. Quantitative Analysis of Samples from Different Origins

These triterpenes showed plenty of bioactivities, and its presence and quantity had a vital influence on the health effect of *M. cocos*. The contents of five compounds were presented as the box-plots with medians (lines in the boxes). For wild fungal samples (Figure S2), Dali (DL) showed smaller amount of poricoic acid A than the other four places (*P* < 0.05). Baoshan (BS) possessed higher content of dehydropachymic acid than the remaining collection locations and greater pachymic acid content than Chuxiong (CX). Compared with DL and BS, Yuxi (YX) had higher concentration of dehydrotrametenolic acid. Pu'er (PE) was significantly different from DL in the concentration of dehydrotrametenolic acid. The cultivated samples from BS were significantly different from those from the other geographical origins in terms of the contents of dehydrotumulosic acid, poricoic acid A, and dehydropachymic acid. Furthermore, for cultivated fungi, both CX and YX were significantly different from DL and PE in dehydrotumulosic acid, DL in dehydropachymic acid, BS, and DL and PE in pachymic acid. These quantitative results of five bioactive analytes gave a valuable reference for differentiating samples derived from different geographical regions and for evaluating the quality of *M. cocos*.

### 3.4. PLS-DA and RF Classification Models of Single Sets

**Figure 6:**
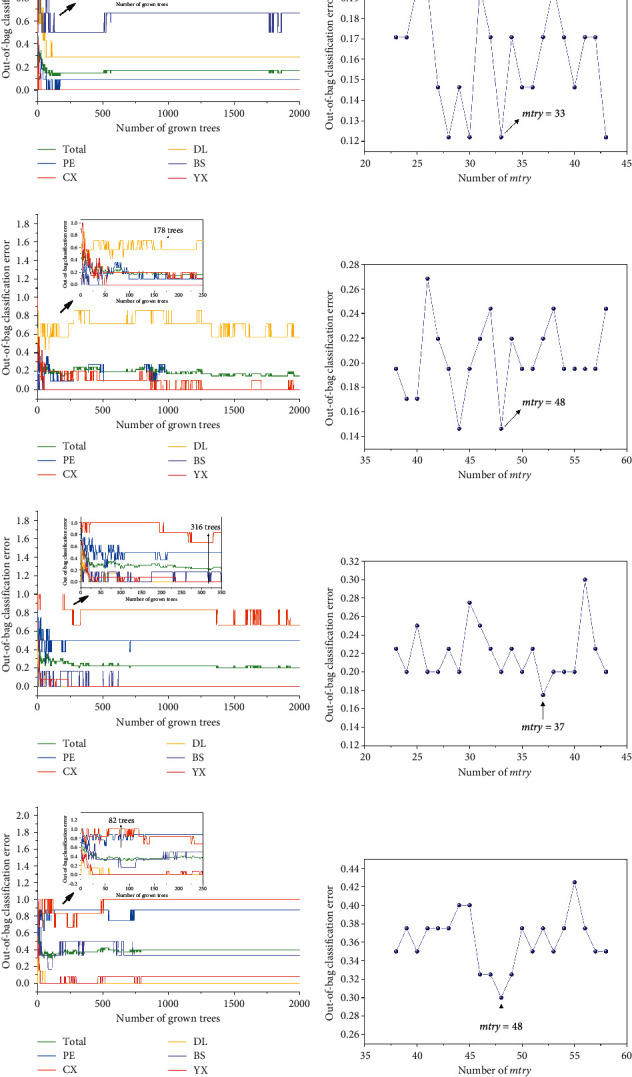
The n_tree_ and *mtry* screening of the random forest models of independent decision making. AB: FTIR of cultivated samples; CD: LC of cultivated samples; EF: FTIR of wild samples; and GH: LC of wild samples.

The selection of parameters was an important step in machine learning methods. The number of latent variables in PLS-DA was defined by 7-fold cross validation by default. In the process of setting up random forest model, two essential parameters were searched based on low OOB error values. Concretely, as for cultivated samples, the optimal n_tree_ and *mtry* were 118 and 33 for FTIR data and 178 and 48 for LC data separately. For wild samples, the final n_tree_ and *mtry* were 316 and 37, respectively, in FTIR model and 82 and 48 in LC model (Figure 6).

The results of independent decision making are shown in Table 1. Both PLS-DA and RF models showed that the cultivated species from different geographical origins could discriminate easily with the total accuracy rates of 95.24% or 100% in validation set. Compared with cultivated samples, the wild ones had lower classification accuracy. Especially, it was difficult to distinguish Class 1 and Class 2, since it showed relatively low efficiency values in calibration and validation sets on the basis of FTIR and LC data. Thus, in order to obtain a better result regarding wild samples, the feasibility of combining the information from FTIR and LC was investigated by means of low-, mid-, and high-level data fusion strategies.

### 3.5. PLS-DA and RF Classification Models of Low-, Mid-, and High-Level Data Fusion

As for low-level strategy, the preprocessed chromatographic and spectral data were straightforward concatenated into a new matrix. In this work, the size of the low-level fusion matrix was equal to (61 × 3484). As described in independent decision making, the optimal PLS-DA and RF models were set up using low-level merged data using suitable parameters (Figure S3). It could be seen from Table 2 that the total accuracy rates of validation set of PLS-DA and RF models (76.19%) were not more than those of single set analysis; therefore, low-level data fusion strategy was unsatisfactory. The main drawback of low-level fusion was that the addition of raw, noisy, and correlated data could worsen the classification results [36]. Hence, one possible reason why low-level fusion was worse than single data analysis was that both LC and FTIR data blocks owned correlated variables (like the information of triterpenes) or noisy.

**Figure 7:**
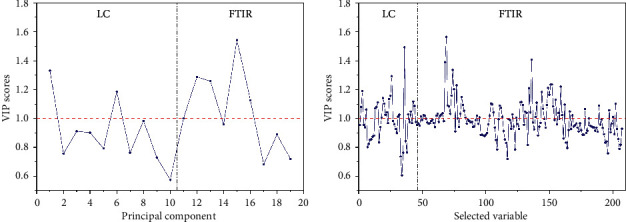
The VIP scores resulting from mid-level PCA (a) and mid-level Boruta (b) PLS-DA models.

In mid-level data fusion, the selected variables by Boruta from LC and FTIR data (green lines in Figure S4) were concatenated into a dataset, and it was called mid-level Boruta. The PCs from LC and FTIR data were combined, which was named as mid-level-PCA. The first ten PCs that described 64.09% of LC variables and first nine PCs that accounted for 79.12% of FTIR variables were extracted. The n_tree_ and *mtry* screening of the random forest models of mid-level-PCA and mid-level Boruta are displayed in Figure S3. Boruta was more efficient than PCA in feature extraction, because mid-level Boruta dataset showed greater efficiency and accuracy of validation set than those of mid-level-PCA one in both PLS-DA and RF models. What is more, the models of mid-level Boruta were superior to the models of low- and high-level data fusion strategies as well as individual techniques due to the highest accuracy of validation set (95.24% and 90.48%). Its PLS-DA model with appropriate accuracy of calibration set (97.50%) was deemed as the best suitable for the geographical identification of wild samples. The variables with VIP scores greater than one (represented by red dashed line) presented in each block of data (Figure 7), indicating that both FTIR and LC were complementary to each other for identifying the origin of the samples.

In the progress of high-level fusion model, the classification votes of calibration and validation set output from each individual model were combined for further majority vote based on four fuzzy aggregation connective operators. As an example (Table S4), the truly class of sample No. 10 belonged to Class 1; however, it was identified as Class 1 in random forest model of FTIR and Class 2 in that of LC, while the voting result based on fuzzy set theory was Class 1. Two types of high-level data fusion were performed, that is, high-level PCA and high-level Boruta. The parameter screening of their random forest models is shown in Figure S5. Random forest models had higher efficiency of validation set for Class 1 and Class 2 than PLS-DA models. However, it was difficult to distinguish Class 1 and Class 2, as always.

Because the accuracy rates of calibration set in PLS-DA models were usually much higher than those of validation set, all of PLS-DA models were validated by permutations test to assess the risk that the current PLS-DA model was spurious. A 30-iteration permutation test was carried out. As could be seen from Figure S6, the regression line of the Q^2^ (predictive squared correlation coefficient) intersected the vertical axis at or below zero; it suggested that the model was not overfitting. The results showed that there was no overfitting in the PLS-DA models.

## 4. Discussion

Under the same condition, comparing the results of cultivated and wild samples geographical identification, it could be found that they showed difference in optimal identification method. For the cultivated ones, it was low-level fusion that preceded mid-level PCA and independent decision making. However, for the wild samples, the performance of individual data and mid-level PCA were better than that of low-level data fusion. And mid-level Boruta of this study was more suitable for the origin identification of wild samples than mid-level PCA. Accordingly, the reason why the mid-level model had better results than the low- and high-level fusion models and independent decision making might be the characters of samples, the feature extraction methods of data fusion, and the final decision methods of high-level data fusion (final decision methods maybe had an influence in the result of high-level data fusion of wild *M. cocos*). What is more, it showed that it was of concern to trace the origin of *M. cocos* from the perspectives of wild and cultivation using multiple data fusion methods. It is worth going to try more data fusion approaches for approximating to an accurate characterization.

## 5. Conclusion

In this study, the geographical traceability of *M. cocos* samples was performed using multiple data fusion methods from the perspectives of wild and cultivation. Low-, mid-level Boruta, mid-level-PCA, high-level Boruta, and high-level PCA data fusion strategies were investigated. Two feature extraction approaches including the selecting variables by Boruta algorithm and producing PCs by the PCA dimension reduction technique were considered in data fusion. The results showed that the geographical traceability of the cultivated was superior to that of the wild. The Baoshan area is suitable for planting *M. cocos* on large noodles.The Yuxi can screen high-quality wild *M. cocos* germplasm resources. The cultivated samples from different collection regions could be easily identified only by FTIR or LC data, while the wild ones could not. In the origin identification of wild samples, Boruta did better than PCA in feature extraction. The PLS-DA and RF models of mid-level Boruta were able to well characterize *M. cocos* and provided a more efficient classification than those of mid-level PCA, low-, high-level PCA, and high-level Boruta data fusion strategies as well as independent decision making, in which the mid-level Boruta PLS-DA model was deemed as the most satisfactory. The mid-level Boruta PLS-DA model provides a reliable method for the identification of the geographical origin of *M. cocos*.

In short, the quality, geographical traceability, and optimal identification methods of cultivated and wild samples were different, and this study also showed the potential of data fusion strategies in the geographical identification of *M. cocos*.

## Figures and Tables

**Table 1:** The classification efficiency and total accuracy rate of independent decision making.

Data source	Model	Calibration set					Total accuracy (%)	Validation set					Total accuracy (%)
		Class 1	Class 2	Class 3	Class 4	Class 5		Class 1	Class 2	Class 3	Class 4	Class 5	
LC-wild	PLS-DA	1	0.91	0.98	1	1	97.50	0.71	0.87	0.94	0.97	0.88	80.95%
	RF	0.50	0.40	0.97	0.91	0.82	70	0.71	0.50	1	0.97	0.86	76.19%
FTIR-wild	PLS-DA	1	1	1	1	1	100	0.66	0.66	1	1	1	80.95%
	RF	0.81	0.40	0.98	1	0.98	82.50	0.69	0.84	1	0.97	1	85.71%
LC-cultivated	PLS-DA	0.95	1	0.99	1	1	97.56	1	1	1	1	1	100%
	RF	0.87	0.93	0.65	1	1	85.37	0.97	1	0.82	1	1	95.24%
FTIR-cultivated	PLS-DA	1	1	1	1	1	100	1	1	1	1	1	100%
	RF	0.93	1	0.85	0.70	1	87.80	1	1	1	1	1	100%

**Table 2:** The classification efficiency and total accuracy rates of different data fusion strategies in wild samples.

Data source	Model	Calibration set					Total accuracy (%)	Validation set					Total accuracy (%)
		Class 1	Class 2	Class 3	Class 4	Class 5		Class 1	Class 2	Class 3	Class 4	Class 5	
Low-level	PLS-DA	1	1	1	1	1	100	0.47	0.71	0.97	0.97	0.97	76.19%
	RF	0.82	0.70	0.98	0.91	0.98	85.00	0.64	0.50	1	0.97	0.97	76.19%
Mid-level PCA	PLS-DA	0.85	0.91	0.98	1	0.98	92.50	0.50	0.84	1	0.97	0.93	80.95%
	RF	0.60	0.71	0.98	0.91	0.86	77.50	0.50	0.49	1	0.97	0.86	71.43%
Mid-level Boruta	PLS-DA	**0.94**	**0.99**	**1**	**1**	**1**	**97.50**	**0.97**	**0.87**	**1**	**1**	**1**	**95.24%**
	RF	0.75	0.68	1	1	1	85	0.84	0.84	1	1	1	90.48%
High-level PCA	PLS-DA	0.98	0.91	1	1	1	97.50	0.49	0.69	0.97	0.97	0.97	76.19%
	RF	0.70	0.80	0.98	0.91	0.92	82.50	0.50	0.87	1	1	0.86	80.95%
High-level Boruta	PLS-DA	1	1	1	1	1	100	0.50	0.69	1	0.91	0.97	76.19%
	RF	0.92	0.90	1	1	1	95	0.71	0.84	1	0.97	0.97	85.71%

## Data Availability

The datasets generated and/or analyzed during the current study are not publicly available because our project is not finished but are available from the corresponding author upon reasonable request.

## Abbreviations

PLS-DA: Partial least squares discriminant analysis
PCA: Principal component analysis
RF: Random forest
LC: Liquid chromatography
UV: Ultraviolet
FTIR: Fourier transform infrared
PCs: Principal components
ATR: Attenuated total reflectance
SG: Savitzky–Golay
VIP: Variable importance for the projection
OOB: Out of bag
n_tree_: Number of trees
TP: True positive
TN: True negative
FP: False positive
FN: False negative
LOQ: Limit of quantification
LOD: Limit of detection
DL: Dali
BS: Baoshan
CX: Chuxiong
YX: Yuxi
PE: Pu'er.

## Supplementary Materials

Supplementary Materials
*Table S1*. The information of *M. cocos* samples. *Table S2*. The limits of detection (LOD) and limits of quantification (LOQ), regression equations, correlation coefficients, and linear ranges of five reference compounds. *Table S3*. The classification efficiency and total accuracy rate of the PLS-DA model processed by SG polynomial second-order derivative. *Table S4*. The class assignation of a sample in high-level data fusion using fuzzy set theory. *Figure S1*. The chromatograms of *M. cocos* at 242 nm and 210 nm. *Note*. Peaks 1–6 are dehydrotumulosic acid, poricoic acid A, 3-epidehydrotumulosic acid, dehydropachymic acid, pachymic acid, and dehydrotrametenolic acid. *Figure S2*. The box-plots of dehydrotumulosic acid, poricoic acid A, dehydropachymic acid, pachymic acid, and dehydrotrametenolic acid in wild (A–E) and cultivated (F–J) *M. cocos* samples from five regions. *Note*. The tick values of vertical are the same. Different letters show significant difference (*P* < 0.5). *Figure S3*. The n_tree_ and *mtry* screening of the random forest models of low-level (AB), mid-level PCA (CD) and mid-level Boruta (EF). *Figure S4*. The variables (under the green lines) selected from LC (left) and FTIR (right) data in mid-level Boruta data fusion, appended with the derivative signals recorded on a sample (red lines). *Figure S5*. The n_tree_ and *mtry* screening of the random forest models regarding high-level data fusion. *Note*. A-B: FTIR of high-level PCA; C-D: LC of high-level PCA; E-F: FTIR of high-level Boruta; G-H: LC of high-level Boruta. *Figure S6*. The permutations plot for mid-level PCA model.
