# Supplementary material for: Comparison of Geographical Traceability of Wild and Cultivated Macrohyporia cocos with Different Data Fusion Approaches
Source: J Anal Methods Chem. 2021 Jul 21;2021:5818999. doi: 10.1155/2021/5818999 (PMC8321747; doi:10.1155/2021/5818999)
Supplement: Supplementary Materials — Table S1. The information of M. cocos samples. Table S2. The limits of detection (LOD) and limits of quantification (LOQ), regression equations, correlation coefficients, and linear ranges of five reference compounds. Table S3. The classification efficiency and total accuracy rate of the PLS-DA model processed by SG polynomial second-order derivative. Table S4. The class assignation of a sample in high-level data fusion using fuzzy set theory. Figure S1. The chromatograms of M. cocos at 242 nm and 210 nm. Note. Peaks 1–6 are dehydrotumulosic acid, poricoic acid A, 3-epidehydrotumulosic acid, dehydropachymic acid, pachymic acid, and dehydrotrametenolic acid. Figure S2. The box-plots of dehydrotumulosic acid, poricoic acid A, dehydropachymic acid, pachymic acid, and dehydrotrametenolic acid in wild (A–E) and cultivated (F–J) M. cocos samples from five regions. Note. The tick values of vertical are the same. Different letters show significant difference (P < 0.5). Figure S3. The ntree and mtry screening of the random forest models of low-level (AB), mid-level PCA (CD) and mid-level Boruta (EF). Figure S4. The variables (under the green lines) selected from LC (left) and FTIR (right) data in mid-level Boruta data fusion, appended with the derivative signals recorded on a sample (red lines). Figure S5. The ntree and mtry screening of the random forest models regarding high-level data fusion. Note. A-B: FTIR of high-level PCA; C-D: LC of high-level PCA; E-F: FTIR of high-level Boruta; G-H: LC of high-level Boruta. Figure S6. The permutations plot for mid-level PCA model. [file 5818999.f1.pdf]

Supplementary Material

Comparison of geographical traceability of wild and cultivated *Macrohyporia cocos* with different data fusion approaches

Li Wang [a, b] Qinqin Wang [c, d], Yuanzhong Wang [*d], Yunmei Wang [*a]

[a] *Quality Standards And Testing Technology Research Institute, Yunnan Academy of Agricultural Sciences, 650205, Kunming, China*

[b] *College of Agronomy and Biotechnology, Yunnan Agricultural University, Kunming 650201, China.*

[c] *The First Affiliated Hospital of Yunnan University of Traditional Chinese Medicine, 650021, Kunming, China*

[d] *Medicinal Plants Research Institute, Yunnan Academy of Agricultural Sciences, 650200, Kunming, China*

[*] Corresponding author telephone: +86 871-65033564 (Yuanzhong Wang).

E-mail addresses: wlwanglidong@126.com (Li Wang), boletus@126.com (Yuanzhong Wang), 1347914675@qq.com (Yunmei Wang).

Table S1.

The information of *M. cocos* samples.

Class	Collection area	Description	Latitude	Longitude	Number
1	Pu'er	Cultivated	N23°25'13.4"	E100°24'15.7"	8
			N23°04'03.5"	E101°58'35.5"	8
		Wild	N24°04'38.4"	E100°47'25.8"	1
			N24°11'33.0"	E101°12'31.1"	1
			N24°10'02.2"	E100°50'38.0"	1
			N23°27'20.2"	E101°43'00.6"	1
			N24°26'59.0"	E100°48'16.5"	1
			N24°03'15.2"	E100°47'18.8"	1
			N24°18'19.0"	E100°57'56.0"	5
			N23°51'10.0"	E100°52'16.4"	1
2	Chuxiong	Cultivated	N24°29'44.6"	E101°34'5.8"	8
			N24°32'08.0"	E101°38'25.2"	8
		Wild	N24°39'57.4"	E101°05'42.7"	1
			N24°52'38.7"	E100°56'32.2"	1
			N25°11'16.5"	E100°53'52.4"	1
			N24°40'48.9"	E101°16'57.0"	1
			N24°53'57.1"	E101°07'37.3"	1
			N24°28'08.3"	E101°32'52.6"	1
			N24°59'22.3"	E100°54'23.0"	1
			N25°01'47.3"	E100°51'21.1"	1
			N24°33'29.4"	E101°26'29.8"	1
			N24°26'24.0"	E101°13'55.0"	1
3	Dali	Cultivated	N25°38'09.8"	E99°7'55.08"	10
		Wild	N25°32'09.8"	E99°41'05.7"	10
4	Baoshan	Cultivated	N24°28'08.5"	E99°30'11.5"	10
		Wild	N24°41'07.8"	E98°36'52.6"	10
5	Yuxi	Cultivated	N24°25'54.7"	E102°31'05.6"	10
		Wild	N23°48'23.2"	E102°51'59.4"	9
			N23°22'25.3"	E101°51'07.7"	10

Table S2.

The limits of detection (LOD) and limits of quantification (LOQ), regression equations, correlation coefficients and linear ranges of five reference compounds.

Components	LOD ($mg \cdot mL^{-1}$)	LOQ ($mg \cdot mL^{-1}$)	Regression equation	r^2	Linear range ($mg \cdot mL^{-1}$)
Dehydrotumulosic acid	0.0002	0.0005	$Y = 11319140.93X + 74475.78$	0.9983	0.005–1.0
Dehydropachymic acid	0.00024	0.0015	$Y = 12988529.78X + 7769.17$	0.9998	0.0024–0.48
Pachymic acid	0.010	0.10	$Y = 7905709.32X + 42996.45$	0.9937	0.010–1.2
Dehydrotrametenolic acid	0.00049	0.002	$Y = 21538210.94X + 107194.87$	0.9993	0.00049–2.4
Poricoic acid A	0.00022	0.00045	$Y = 22522136.84X + 42388.59$	0.9999	0.00022–1.1

Table S3.

The classification efficiency and total accuracy rate of the PLS-DA model processed by SG polynomial second-derivative.

Data source	Pretreatment	Calibration set					Total accuracy (%)	Validation set					Total accuracy (%)
		Class 1	Class 2	Class 3	Class 4	Class 5		Class 1	Class 2	Class 3	Class 4	Class 5	
LC-wild	Raw	0.35	0.87	1	0.99	0.89	77.50%	0	0.97	1	1	0.89	80.95%
	Processed	1	0.91	0.98	1	1	97.50%	0.71	0.87	0.94	0.97	0.88	80.95%
FTIR-wild	Raw	0	0.55	0.87	0.40	0.73	50%	0	0.66	0.91	0.49	0.70	47.62%
	Processed	1	1	1	1	1	100%	0.66	0.66	1	1	1	80.95%
LC-cultivated	Raw	0.92	0.98	0.76	1	0.99	90.24%	0.84	0.97	0	1	0.97	80.95%
	Processed	0.95	1	0.99	1	1	97.56%	1	1	1	1	1	100%
FTIR-cultivated	Raw	0.91	1	0.53	0.41	0.92	75.61%	0.90	1	1	0	0.97	80.95%
	Processed	1	1	1	1	1	100%	1	1	1	1	1	100%

Table S4.

The class assignation of a sample in high-level data fusion using fuzzy set theory.

Class 1, NO. 10	Tree vote fractions of RF model					Individual decision	Ensemble decision
	Class 1	Class 2	Class 3	Class 4	Class 5		
FTIR-Boruta	0.55	0.17	0.04	0.06	0.17	Class 1	
LC-Boruta	0.23	0.36	0.09	0.16	0.17	Class 2	
Minimum	0.23	0.17	0.04	0.06	0.17		Class 1
Maximum	0.55	0.36	0.09	0.16	0.17		Class 1
Product	0.13	0.06	0.00	0.01	0.03		Class 1
Average	0.39	0.26	0.07	0.11	0.17		Class 1
Majority vote	√ √ √ √						Class 1

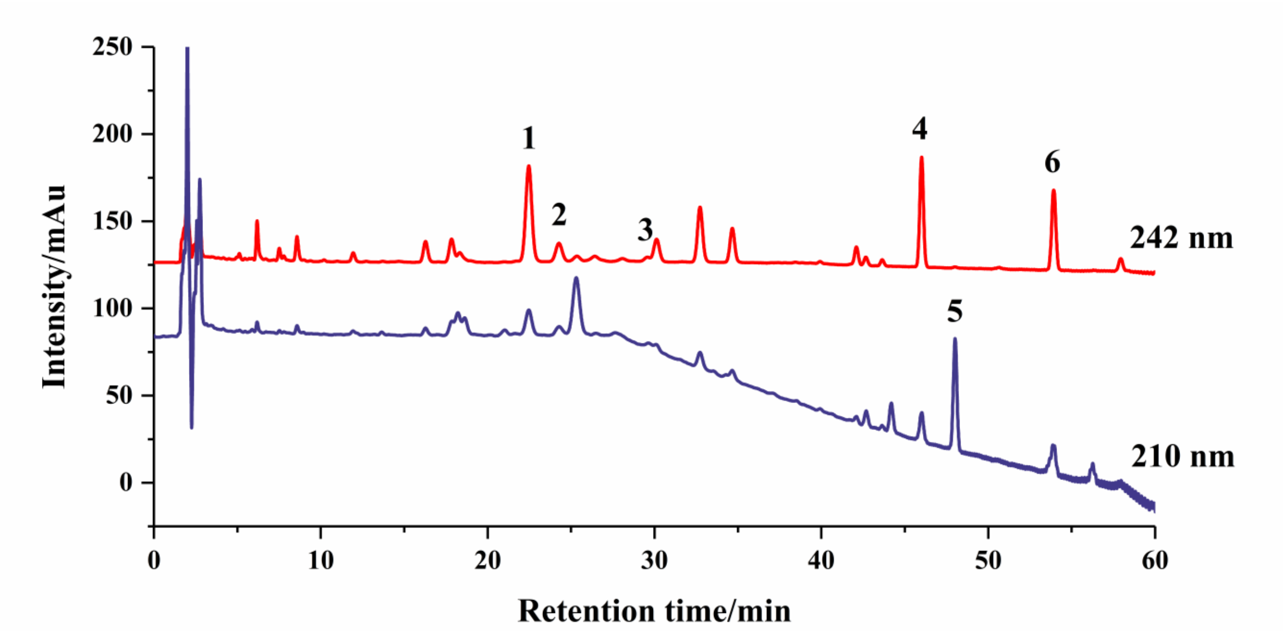

Figure S1. The chromatograms of *M. cocos* at 242 nm and 210 nm. Note: Peak 1-6 are dehydrotumulosic acid, poricoic acid A, 3-epidehydrotumulosic acid, dehydropachymic acid, pachymic acid and dehydrotrametenolic acid.

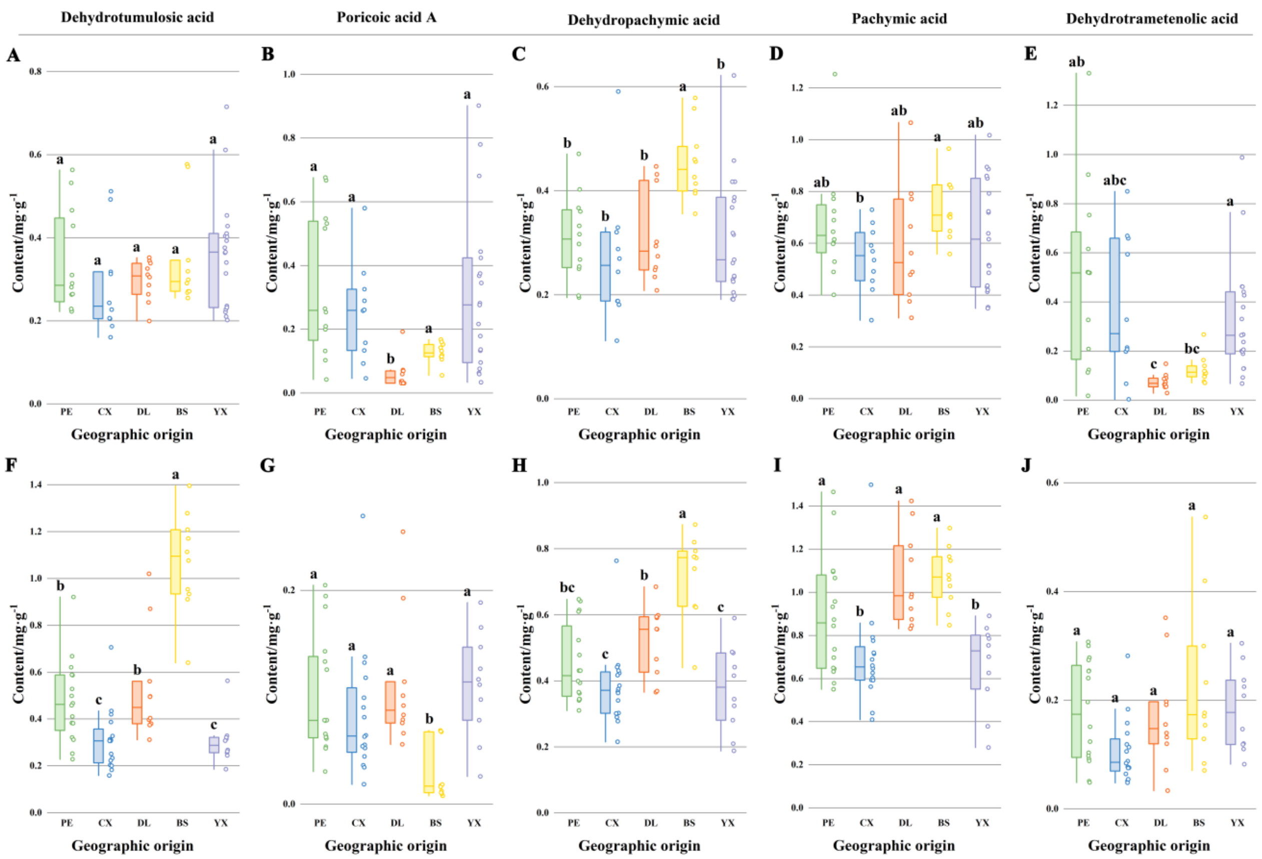

Figure S2. The box-plots of dehydrotumulosic acid, poricoic acid A, dehydropachymic acid, pachymic acid and dehydrotrametenolic acid in wild (A-E) and cultivated (F-J) *M. cocos* samples from five regions. Note: The tick values of vertical are the same; Different letters show significant difference ($P < 0.05$).

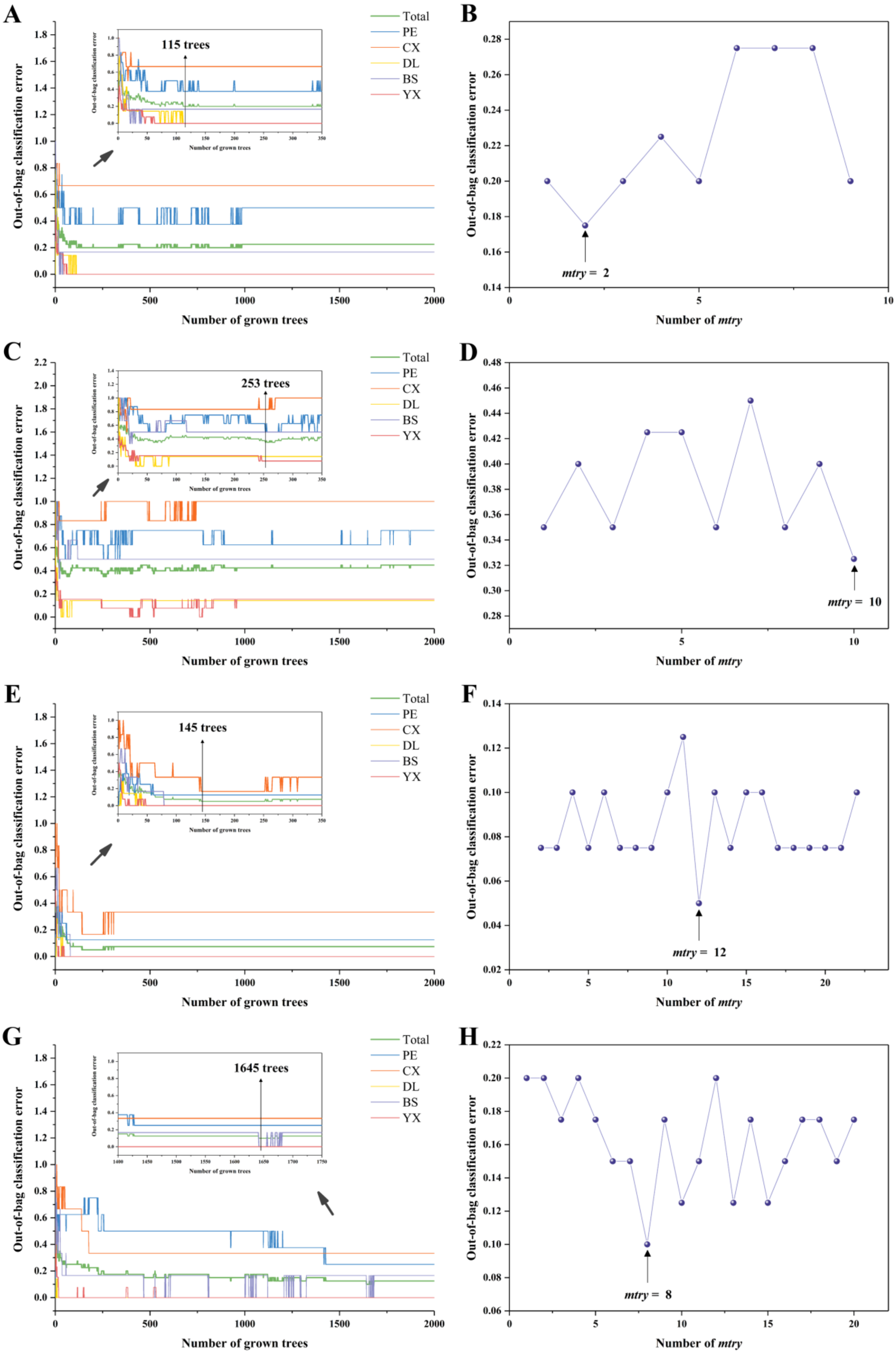

Figure S3. The n_{tree} and *mtry* screening of the random forest models of low-level (AB), Mid-level-PCA (CD) and Mid-level-Boruta (EF).

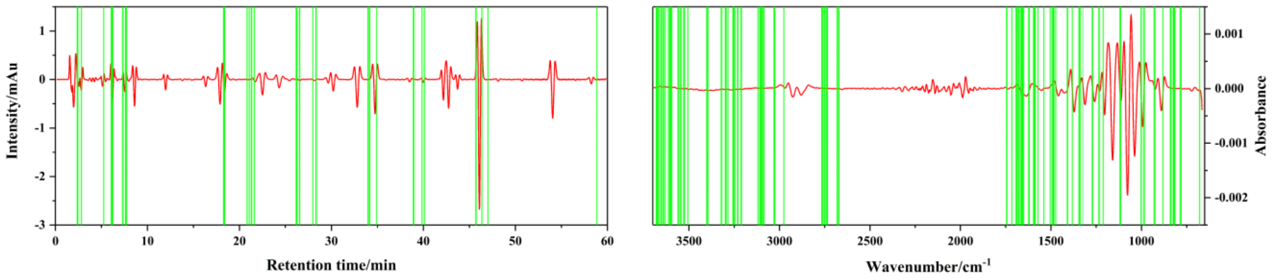

Figure S4. The variables (under the green lines) selected from LC (left) and FTIR (right) data in Mid-level-Boruta data fusion, appended with the derivative signals recorded on a sample (red lines).

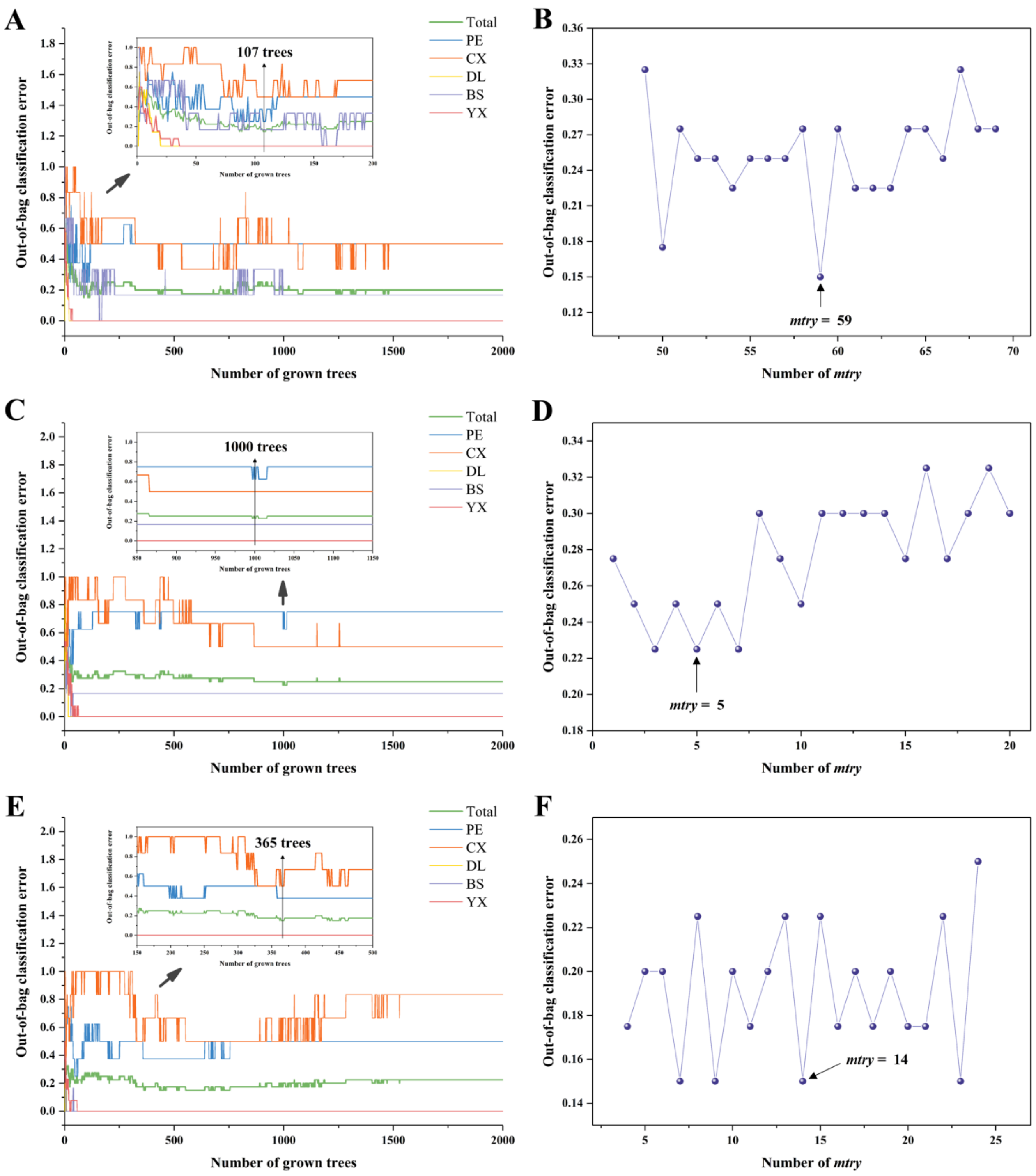

Figure S5. The n_{tree} and *mtry* screening of the random forest models regarding high-level data fusion. Note: A-B: FTIR of High-level-PCA; C-D: LC of High-level-PCA; E-F: FTIR of High-level-Boruta; G-H: LC of High-level-Boruta.

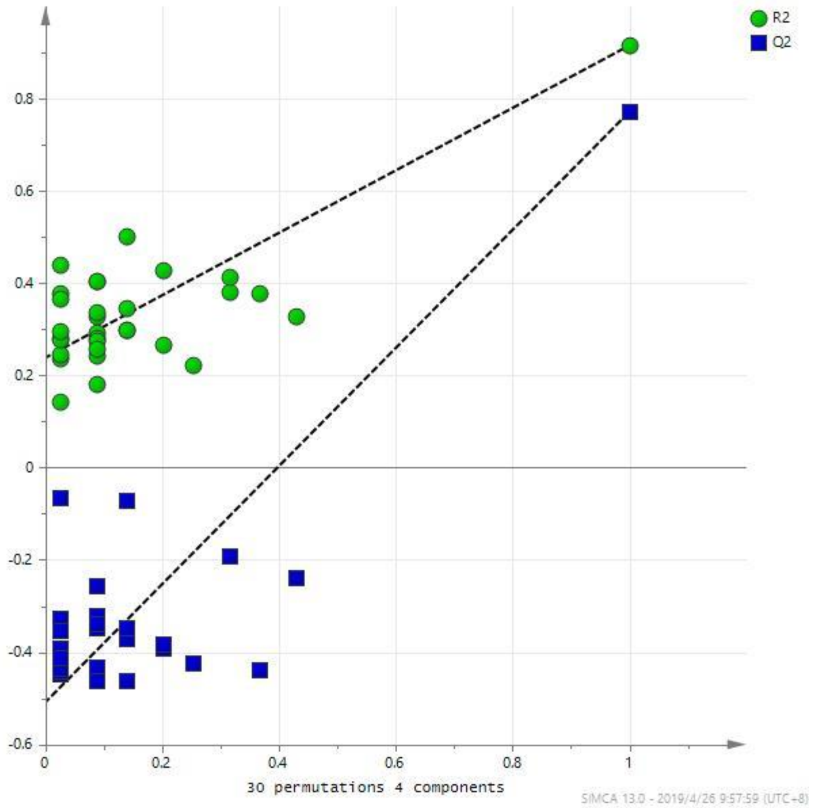

Figure S6. The permutations plot for Mid-level-PCA model.
